# Supplementary material for: PD-1/PD-L1 Inhibitors plus Chemotherapy Versus Chemotherapy Alone for Resectable Non-Small Cell Lung Cancer: A Systematic Review and Meta-Analysis of Randomized Controlled Trials
Source: Cancers (Basel). 2023 Oct 26;15(21):5143. doi: 10.3390/cancers15215143 (PMC10648147; doi:10.3390/cancers15215143)
Supplement: Supplementary file 1 [file cancers-15-05143-s001.zip › cancers-2607980-supplementary.pdf]

Supplementary Material

Supplementary Table S1 Inclusion and exclusion criteria of included studies.

Supplementary Table S2 Search strategies.

Supplementary Table S3 Additional baseline characteristics of included studies.

Supplementary Table S4 Treatment regimens from the randomized controlled trials included in this systematic review and meta-analysis.

Supplementary Figure S1 Any grade of adverse events. **A** Arthralgia. **B** Increased alanine aminotransferase. **C** Hypothyroidism. Comparison between programmed cell death protein 1 (PD-1)/programmed death-ligand 1 (PD-L1) inhibitors plus chemotherapy and chemotherapy alone in patients with resectable stage non-small cell lung cancer. CI, confidence interval; MH, Mantel–Haenszel.

Supplementary Figure S2 Any grade of adverse events. **A** Rash. **B** Fatigue. **C** Pruritus. Comparison between programmed cell death protein 1 (PD-1)/programmed death-ligand 1 (PD-L1) inhibitors plus chemotherapy and chemotherapy alone in patients with resectable stage non-small cell lung cancer. CI, confidence interval; MH, Mantel–Haenszel.

Supplementary Figure S3 Any grade of adverse events. **A** Diarrhea. **B** Nausea. **C** Decreased appetite. Comparison between programmed cell death protein 1 (PD-1)/programmed death-ligand 1 (PD-L1) inhibitors plus chemotherapy and chemotherapy alone in patients with resectable stage non-small cell lung cancer. CI, confidence interval; MH, Mantel–Haenszel.

Supplementary Figure S4 Any grade of adverse events. **A** Anemia. **B** Constipation. **C** Neutrophil count decreased. Comparison between programmed cell death protein 1 (PD-1)/programmed death-ligand 1 (PD-L1) inhibitors plus chemotherapy and chemotherapy alone in patients with resectable stage non-small cell lung cancer. CI, confidence interval; MH, Mantel–Haenszel.

Supplementary Figure S5 Grade ≥3 adverse events. **A** Fatigue. **B** Diarrhea. **C.** Increased alanine aminotransferase. Comparison between programmed cell death protein 1 (PD-1)/programmed death-ligand 1 (PD-L1) inhibitors plus chemotherapy and chemotherapy alone in patients with resectable stage non-small cell lung cancer. CI, confidence interval; MH, Mantel–Haenszel.

Supplementary Figure S6 Grade ≥3 adverse events. **A** Neutrophil count decreased. **B** Rash. **C** Decreased appetite. Comparison between programmed cell death protein 1 (PD-1)/programmed death-ligand 1 (PD-L1) inhibitors plus chemotherapy and chemotherapy alone in patients with resectable stage non-small cell lung cancer. CI, confidence interval; MH, Mantel–Haenszel.

Supplementary Figure S7 Leave-one-out sensitivity analyses. **A** Overall survival. **B** Event-free survival and disease-free survival. CI, confidence interval; HR, hazard ratio; IV, inverse variance; PD-1, programmed cell death protein 1; PD-L1, programmed death-ligand 1.

Table S1 Inclusion and exclusion criteria of included studies.

**Study**	**Inclusion Criteria**	**Exclusion Criteria**
CheckMate 816<br>2022	1. Early stage IB-IIIA, operable non-small cell lung cancer, confirmed in tissue<br>2. Lung function capacity capable of tolerating the proposed lung surgery<br>3. Eastern Cooperative Oncology Group (ECOG) Performance Status of 0-1<br>4. Available tissue of primary lung tumor	1. Presence of locally advanced, inoperable or metastatic disease<br>2. Participants with active, known or suspected autoimmune disease<br>3. Prior treatment with any drug that targets T cell co-stimulation pathways (such as checkpoint inhibitors)
IMpower010<br>2021	Inclusion Criteria for Enrollment Phase<br>1. Eastern Cooperative Oncology Group (ECOG) performance status of 0 or 1<br>2. Histological or cytological diagnosis of Stage IB (tumors greater than or equal to (>/=) 4 centimeters (cm))-IIIA (T2-3 N0, T1-3 N1, T1-3 N2, T4 N0-1) NSCLC (per the Union Internationale Contre le Cancer staging system (UICC)/American Joint Committee on Cancer staging system (AJCC) staging system, 7th edition; Detterbeck et al. 2009)<br>3. Participants must have had complete resection of NSCLC 4-12 weeks (>/=28 days and less than or equal to (</=) 84 days) prior to enrollment and must be adequately recovered from surgery<br>4. If mediastinoscopy was not performed preoperatively, it is required that, at a minimum, mediastinal lymph node systematic sampling will have occurred. Systematic sampling is defined as removal of at least one representative lymph node at specified levels. MLND	Exclusion Criteria for Enrollment Phase<br>1. Illness or condition that may interfere with a participant's capacity to understand, follow, and/or comply with study procedures<br>2. Pregnant and lactating women<br>3. Treatment with prior systemic chemotherapy: Chemotherapy for early stage of malignancy with curative intent, provided that the last dose received was more than 5 years prior to enrollment and low-dose chemotherapy for non-malignant conditions may be allowed upon approval by the Medical Monitor<br>4. Hormonal cancer therapy or radiation therapy as prior cancer treatment within 5 years before enrollment<br>5. Treatment with any other investigational agent with therapeutic intent within 28 days prior to enrollment<br>6. Participants with hearing impairment<br>7. Known sensitivity to any component of the chemotherapy regimen the participant will be assigned to, or to mannitol

	entails resection of all lymph nodes at those same levels. For a right thoracotomy, sampling or MLND is required at levels 4 and 7 and for a left thoracotomy, levels 5 and/or 6 and 7. Exceptions will be granted if there is clear documentation in the operative report or in a separately submitted addendum by the surgeon of exploration of the required lymph node areas, and the participant will be considered eligible if no lymph nodes are found in those areas; if participants have documented N2 disease in one level (per the UICC/AJCC staging system, 7th edition; Detterbeck et al. 2009), not all levels need to be sampled; if the preoperative staging imaging results (contrast computed tomography (CT) and positron emission tomography (PET) scans) do not suggest evidence of disease in the mediastinum, the participant will be considered eligible if N2 nodal sampling is not performed per surgeon's decision<br>5. Eligible to receive a cisplatin-based chemotherapy regimen<br>6. Adequate hematologic and end-organ function<br>7. Women who are not postmenopausal (>/=12 months of non-therapy-induced amenorrhea) or surgically sterile must have a negative serum pregnancy test result within 14 days prior to initiation of cisplatin-based chemotherapy<br><br>Inclusion Criteria for Randomized Phase: Women who are not postmenopausal (>/=12 months of non-therapy-induced amenorrhea) or surgically sterile must have a negative serum pregnancy test result within 14 days prior to initiation of atezolizumab or BSC	8. Prior treatment with cluster of differentiation (CD) 137 (CD137) agonists or immune checkpoint blockade therapies, anti-programmed death-1 (PD-1), and anti programmed death ligand 1 (PD-L1) therapeutic antibodies<br>9. Malignancies other than NSCLC within 5 years prior to randomization, with the exception of those with a negligible risk of metastasis or death (e.g., expected 5-year OS greater than (>) 90 percent (%)) treated with expected curative outcome (such as adequately treated carcinoma in situ of the cervix, basal or squamous cell skin cancer, localized prostate cancer treated surgically with curative intent, ductal carcinoma in situ treated surgically with curative intent))<br>10. History of severe allergic, anaphylactic, or other hypersensitivity reactions to chimeric or humanized antibodies or fusion proteins<br>11. Known hypersensitivity to biopharmaceuticals produced in Chinese hamster ovary cells or any component of the atezolizumab formulation<br>12. History of autoimmune disease, including but not limited to myasthenia gravis, myositis, autoimmune hepatitis, systemic lupus erythematosus, rheumatoid arthritis, inflammatory bowel disease, vascular thrombosis associated with antiphospholipid syndrome, Wegener's granulomatosis, Sjögren's syndrome, Guillain–Barré syndrome, multiple sclerosis, vasculitis, or glomerulonephritis<br>13. Positive test for human immunodeficiency virus (HIV)<br>14. Participants with active hepatitis B (chronic or acute, defined as having a positive hepatitis B surface antigen (HBsAg) test at screening) or hepatitis C

		15. Active tuberculosis<br>16. Significant cardiovascular disease, such as New York Heart Association cardiac disease (Class II or greater), myocardial infarction, or cerebrovascular accident within the previous 3 months, unstable arrhythmias, or unstable angina<br>17. History of idiopathic pulmonary fibrosis, organizing pneumonia (e.g., bronchiolitis obliterans), drug-induced pneumonitis, idiopathic pneumonitis, or evidence of active pneumonitis on screening chest CT scan<br>18. Prior allogeneic bone marrow transplantation or solid organ transplant<br>19. Any other diseases, metabolic dysfunction, physical examination finding, or clinical laboratory finding giving reasonable suspicion of a disease or condition that contraindicates the use of an investigational drug or that may affect the interpretation of the results or render the participant at high risk of treatment complications<br>20. Known tumor PD-L1 expression status as determined by an immunohistochemistry (IHC) assay from other clinical studies (e.g., participants whose PD-L1 expression status was determined during screening for entry into a study with anti-PD-1 or anti-PD-L1 antibodies but were not eligible are excluded)<br><br>Specific Exclusions for Pemetrexed Treatment<br>1. Participants with squamous cell histology<br><br>Exclusion Criteria for Randomized Phase<br>1. Signs or symptoms of infection within 14 days prior to randomization (severe infection within 28 days prior to randomization), including but not limited to

		hospitalization for complications of infection, bacteremia, or severe pneumonia<br>2. Received therapeutic oral or intravenous (IV) antibiotics within 14 days prior to randomization<br>3. Major surgical procedure within 28 days prior to randomization or anticipation of need for a major surgical procedure during the course of the study<br>4. Administration of a live, attenuated vaccine within 4 weeks prior to initiation of study treatment or anticipation that such a live attenuated vaccine will be required during the study<br>5. Treatment with systemic immunostimulatory agents (including but not limited to interferons or interleukin-2) within 4 weeks or 5 half-lives of the drug, whichever is longer, prior to randomization; prior treatment with cancer vaccines is allowed<br>6. Treatment with systemic corticosteroids or other immunosuppressive medications (including but not limited to prednisone, dexamethasone, cyclophosphamide, azathioprine, methotrexate, thalidomide, and anti-tumor necrosis factor (anti-TNF) agents) within 14 days prior to randomization
KEYNOTE-671 2023	1. Have previously untreated and pathologically confirmed resectable Stage II, IIIA, or IIIB (N2) NSCLC.<br>2. If male, must agree to use contraception or practice abstinence as well as refrain from donating sperm during the treatment period and for the time needed to eliminate each study intervention after the last dose of study intervention.<br>3. If female, may participate if not pregnant or breastfeeding,	1. Has one of the following tumor locations/types: 1) NSCLC involving the superior sulcus; 2) large cell neuro-endocrine cancer (LCNEC); or 3) sarcomatoid tumor.<br>2. Has a history of (non-infectious) pneumonitis/interstitial lung disease that required steroids or has current pneumonitis/interstitial lung disease that requires steroids.

	and at least one of the following conditions apply: 1) not a woman of childbearing potential (WOCBP); or 2) a WOCBP who agrees to follow contraceptive guidance during the treatment period and for the time needed to eliminate each study intervention after the last dose of study intervention and agrees not to donate eggs (ova, oocytes) to others or freeze/store for her own use for the purpose of reproduction during this period.<br>4. Have available formalin-fixed paraffin embedded (FFPE) tumor tissue sample blocks for submission. If blocks are not available, have unstained slides for submission for central programmed death-ligand 1 (PD-L1) testing.<br>5. Have an Eastern Cooperative Oncology Group (ECOG) performance status of 0 to 1 within 10 days of randomization.<br>6. Have adequate organ function.	3. Has an active infection requiring systemic therapy.<br>4. Has had an allogenic tissue/sold organ transplant.<br>5. Has a known severe hypersensitivity (≥ Grade 3) to pembrolizumab, its active substance, and/or any of its excipients.<br>6. Has a known severe hypersensitivity (≥ Grade 3) to any of the study chemotherapy agents and/or to any of their excipients.<br>7. Has an active autoimmune disease that has required systemic treatment in past 2 years.<br>8. Has a known history of human immunodeficiency virus (HIV) infection.<br>9. Has a known history of Hepatitis B or Hepatitis C.<br>10. Has a known history of active tuberculosis.<br>11. Has a history or current evidence of any condition, therapy, or laboratory abnormality that might confound the results of the trial, interfere with the participant's participation for the full duration of the trial, or is not in the best interest of the participant to participate.<br>12. Has known psychiatric or substance abuse disorders that would interfere with cooperating with the requirements of the trial.<br>13. Has received prior therapy with an anti-PD-1, anti-PD-L1, or anti-PD-L2 agent or with an agent directed to another co-inhibitory T-cell receptor.<br>14. Has received prior systemic anti-cancer therapy including investigational agents for the current malignancy prior to randomization/allocation.<br>15. Has received prior radiotherapy within 2 weeks of start of trial treatment.<br>16. Has received a live vaccine within 30 days prior to the first dose of trial drug.

		17. Is currently participating in or has participated in a trial of an investigational agent or has used an investigational device within 4 weeks prior to the first dose of trial treatment.<br>18. Has a diagnosis of immunodeficiency or is receiving either systemic steroid therapy or any other form of immunosuppressive therapy within 7 days prior the first dose of trial drug.<br>19. Has a known additional malignancy that is progressing or requires active treatment within the past 5 years.
NADIM II 2023	1. Previously untreated patients with histologically or cytologically documented NSCLC who present stage IIIA disease (according to 8th version of the International Association for the Study of Lung Cancer Staging Manual in Thoracic Oncology) and also, potentially resectable locally advanced NSCLC patients' stage IIIB with T3N2 disease according to 8th edition can be included.<br>a. PET/CT including IV contrast (CT of diagnostic quality) will be performed at baseline (28 days +10 before randomization)<br>2. Tumor should be considered resectable before study entry by a multidisciplinary team<br>3. ECOG (Performance status) 0-1<br>4. Screening laboratory values must meet the following criteria and should be obtained within 14 days prior to randomization.<br>i. Neutrophils ≥ 1500×109/L ii. Platelets ≥ 100 x×109/L iii. Hemoglobin > 9.0 g/dL iv. Serum creatinine ≤ 1.5 x ULN or creatinine clearance (CrCl) ≥ 40 mL/min v. AST/ALT ≤ 3 x ULN vi. Total Bilirubin ≤ 1.5 x ULN (except subjects with Gilbert Syndrome, who can have total bilirubin < 3.0	1. All patients carrying activating mutations in the TK domain of EGFR or any variety of alterations in the ALK gene.<br>2. Patients with active, known, or suspected autoimmune disease. Subjects with vitiligo, type I diabetes mellitus, residual hypothyroidism due to autoimmune thyroiditis only requiring hormone replacement or unexpected conditions of recurrence in the absence of an external trigger are allowed to be included.<br>3. Patients with a condition requiring systemic treatment with either corticosteroids (>10 mg daily prednisone equivalent) or other immunosuppressive medications within 14 days of randomization. Inhaled or topical steroids, and adrenal replacement steroid doses > 10 mg daily prednisone equivalent, are permitted in the absence of active autoimmune disease.<br>4. Patients with a history of interstitial lung disease cannot be included if they have symptomatic ILD (Grade 3-4) and/or poor lung function. In case of doubt, please contact trial team.<br>5. Patients with other active malignancy requiring

	mg/dL) vii. The patients need to have a forced expiratory volume (FEV1) ≥ 1.2 liters or >40% predicted value viii. INR/APTT within normal limits<br>5. All patients are notified of the investigational nature of this study and signed a written informed consent in accordance with institutional and national guidelines, including the Declaration of Helsinki prior to any trial-related intervention<br>6. Patients aged > 18 years<br>7. Women of childbearing potential, including women who had their last menstrual period in the last 2 years, must have a negative serum or urine pregnancy test within 7 days before randomization.<br>8. All sexually active men and women of childbearing potential must use an effective contraceptive method (two barrier methods or a barrier method plus a hormonal method) during the study treatment and for a period of at least 12 months following the last administration of trial drugs<br>9. Patient capable of proper therapeutic compliance and accessible for correct follow-up<br>10. Measurable or evaluable disease (according to RECIST 1.1 criteria)	concurrent intervention and/or concurrent treatment with other investigational drugs or anti-cancer therapy<br>6. Patients with previous malignancies (except non-melanoma skin cancers, and the following in situ cancers: bladder, gastric, colon, endometrial, cervical/dysplasia, melanoma, or breast) are excluded unless a complete remission was achieved at least 2 years prior to study entry AND no additional therapy is required during the study period.<br>7. Any medical, mental, or psychological condition which in the opinion of the investigator would not permit the patient to complete the study or understand the patient information<br>8. Patients who have had prior treatment with an anti-PD-1, anti-PD-L1, anti-PD-L2, anti-CTLA-4 antibody, or any other antibody or drug specifically targeting T-cell costimulation or immune checkpoint pathways<br>9. Patients with positive test for hepatitis B virus surface antigen (HBV sAg) or hepatitis C virus ribonucleic acid (HCV antibody) indicating acute or chronic infection<br>10. Patients with known history of testing positive for human immunodeficiency virus (HIV) or known acquired immunodeficiency syndrome (AIDS)<br>11. Patients with history of allergy to study drug component excipients<br>12. Women who are pregnant or in the period of breastfeeding<br>13. Sexually active men and women of childbearing potential who are not willing to use an effective contraceptive method during the study
NEOTORCH	1. Having sufficient understanding of this study and being	1. Presence of locally advanced, unresectable or metastatic

2023	willing to sign the informed consent form (ICF)<br>2. Aged 18-70 years, male or female<br>3. Treatment-naive, histologically confirmed resectable, stage II, IIIA, IIIB (N2) (AJCC staging system, version 8) NSCLC; cTNM stage can be confirmed through PET-CT or pathological biopsy; resectable stage II non-small cell lung cancer is defined as eligible for radical resection evaluated by a qualified thoracic surgeon; resectable stage III is defined as the resectable and potential resectable according to the Chinese expert consensus on the multidisciplinary diagnosis and treatment for stage III non-small cell lung cancer (2019) in which resectable includes IIIA(N0-1), partial N2 with single-station mediastinal lymph node metastasis and the short diameter of lymph node <2 cm, partial T4 (satellite nodules in the adjacent lobe) N1 and potential resectable includes partial stage IIIA and IIIB with the short diameter of single-station N2 mediastinal lymph node<3 cm, other potentially resectable T3 or T4 central tumor; for any suspected lesions which could change the TNM stage, such as contralateral mediastinal lymph node, supraclavicular lymph mode, solid/sub-solid pulmonary node, and non-isolated ground glass opacity (GGO), pathological confirmation is strongly recommended.<br>4. Measurable lesions based on the response evaluation criteria in solid tumors version 1.1<br>5. Tumor tissue specimens available for pathological diagnosis, detection of PD-L1 expression and biomarkers prior to randomization (the tumor tissue specimens must be freshly obtained or archived samples within 3 months prior to enrollment; tumor tissue specimens must be the samples of histological category, including but not limited to the tissue punctured by core needle and hollow needle, tissue	disease; unresectable includes the unresectable definition in the Chinese expert consensus on the multidisciplinary diagnosis and treatment for stage III non-small cell lung cancer (2019), including partial stage IIIA and IIIB and all the stage IIIC; N2: single-station mediastinal lymph node metastasis with short diameter ≥3cm; N2: multiple-station mediastinal lymph node metastasis with lymph node fusion and the short diameter of lymph node ≥2cm on CT; ALL the N3; T4: invading esophagus, heart, aorta<br>2. NSCLC involving superior sulcus, large cell neuroendocrine carcinoma (LCNEC), sarcomatoid tumor<br>3. Participants with known EGFR sensitive mutations or ALK translocation; EGFR and ALK mutation status needs to be identified for the subjects with non-squamous cell carcinoma<br>4. Previous treatment with systemic antitumor therapy for early NSCLC, including investigational product<br>5. History of (non-infectious) pneumonitis/interstitial lung disease requiring steroid treatment, or ongoing pneumonitis/interstitial lung disease requiring steroid treatment<br>6. Active tuberculosis<br>7. Active infection requiring systemic treatment<br>8. Subjects with any known or suspected autoimmune disorder or immunodeficiency, with the following exceptions: hypothyroidism, hormone therapy is not needed, or well-controlled at physiological dose; controlled type I diabetes<br>9. Uncontrolled active hepatitis B (defined as positive hepatitis B surface antigen (HBsAg) in screening period

	acquired by bronchoscopic clamp, surgically resected samples; the samples acquired by fine needle puncture and bronchial brushing are not acceptable)<br>6. ECOG score 0-1<br>7. Good organ function<br>8. Being willing and able to comply with the visits, treatment plan, laboratory examinations and other study procedures scheduled in the study<br>9. Pulmonary function test being able to withstand the planned pneumonectomy evaluated by surgeons; women of childbearing potential must undergo serum pregnancy test within 3 hours prior to the first dose and the result must be negative. Female subjects of childbearing potential and male subjects whose partners are women of childbearing potential must agree to use highly effective contraceptive methods during the study period and within 180 days after the last dose of study drug.<br>10. Women of childbearing potential must undergo serum pregnancy test within 3 days prior to the first dose and the result must be negative. Female subjects of childbearing potential and male subjects whose partners are women of childbearing potential must agree to use highly effective contraceptive methods during the study period and within 180 days after the last dose of study drug	with HBV-DNA detected higher than the upper limit of normal at the clinical laboratory of the study center); the subjects with HBV-DNA assay <500 IU/mL within 28 days prior to randomization who have received local standard antiviral therapy for at least 14 days and are willing to receive antiviral therapy continuously during the study can be enrolled; active hepatitis C (defined as positive hepatitis C surface antibody (HCsAb) in screening period and positive HCV-RNA)<br>10. Known human immunodeficiency virus (HIV) infection (known positive HIV antibody)<br>11. Vaccination with live vaccine within 30 days prior to the first dose, including but not limited to the following: parotitis, rubella, measles, varicella/ herpes zoster (varicella), yellow fever, Rabies, Bacille Calmette–Guérin (BCG) and typhoid vaccine (inactivated virus vaccine allowed)<br>12. ≥ Grade 2 peripheral neuropathy<br>13. Previous use of PD-1/PD-L1 agent or the drug acting on another targeted T cell receptor (e.g., CTLA-4, OX-40)<br>14. Severe allergic reaction to other monoclonal antibodies<br>15. History of serious allergy to Pemetrexed, paclitaxel or docetaxel, cisplatin, carboplatin or its preventive medications<br>16. Known serious or uncontrolled pre-existing diseases, including but not limited to cardiovascular events with hemodynamic instability, symptomatic cerebrovascular events, and hepatic cirrhosis above Child-Pugh A within 6 months<br>17. History or current evidence of any disease, therapy or abnormal laboratory examination that may confuse the study results, interfere with subject's participation in the

		full course of the study, or not meet the best interest of subject's participation in the study, as judged by investigators<br>18. Other malignant tumors within 5 years prior to the first dose, except non-small cell lung cancer. The malignant tumors with negligible risk of metastasis or death (e.g., expected disease-free survival > 5 years) and expected to achieve radical outcomes after treatment (e.g., sufficiently treated carcinoma in situ of cervix, basal or squamous cell skin cancer, ductal carcinoma in situ treated for radical surgery) can be excluded.
PEARLS/KEYNOTE-091 2022	1. Pathological diagnosis of NSCLC confirmed at surgery, any histology. Participants with two synchronous primary non-small cell lung cancers are excluded from the study<br>2. Union for International Cancer Control (UICC) v7 Stage IB with T ≥ 4 cm, II-IIIA NSCLC after complete surgical resection with resection margins proved microscopically free of disease (R0). Carcinoma in situ can be present at the bronchial margin<br>3. Available tumor sample obtained at surgical resection for programmed cell death ligand-1 (PD-L1) Immunohistochemistry (IHC) expression assessment<br>4. Eastern Cooperative Oncology Group (ECOG) Performance Status 0-1<br>5. Adequate organ function performed within 10 days of treatment initiation<br>6. Female participants of childbearing potential must have a negative urine or serum pregnancy test at screening (within 72 hours of first infusion of study medication). If the urine test cannot be confirmed as negative, a serum pregnancy test will be required. The serum pregnancy test must be	1. Evidence of disease at clinical examination and/or baseline radiological assessment as documented by contrast enhanced chest/upper abdomen CT scan, brain CT/MRI and clinical examination<br>2. More than 4 cycles of adjuvant therapy<br>3. Prior treatment with anti-programmed cell death (anti-PD)-1, anti-PD ligand-1/2, anti-CD137, or cytotoxic T-lymphocyte-associated protein 4 (CTLA-4) modulators or any other immune-modulating agents<br>4. Live vaccine within 30 days prior to the first infusion of study treatment<br>5. Current participation or treatment with an investigational agent or use of an investigational device within 4 weeks of the first infusion of study treatment<br>6. History of Human Immunodeficiency Virus (HIV) (known HIV 1/2 antibodies positive). No known active Hepatitis B or C<br>7. Chronic use of immunosuppressive agents and/or systemic corticosteroids or any use in the last 3 days prior to the first infusion of study treatment

	negative for the participant to be eligible<br>7. Female participants of childbearing potential must be willing to use 2 methods of birth control or be surgically sterile, or abstain from heterosexual activity starting with the first infusion of study treatment through 120 days after the last infusion of study treatment<br>8. Female participants who are breast feeding must discontinue nursing prior to the first infusion of study medication and until 120 days after the last infusion study treatment<br>9. Male participants must agree to use an adequate method of contraception starting with the first infusion of study treatment through 120 days after the last infusion of study treatment<br>10. Absence of severe comorbidities that in the opinion of the investigator might hamper the participation in the study and/or the treatment administration<br>11. No prior or planned neo-adjuvant or adjuvant radiotherapy and/or neo-adjuvant chemotherapy for the current malignancy is allowed	8. History of interstitial lung disease or (non-infectious) pneumonitis that required oral or IV steroids (other than COPD exacerbation) or current pneumonitis<br>9. Active autoimmune disease that has required systemic treatment in past 2 years<br>10. History of a hematologic or primary solid tumor malignancy, unless in remission for at least 5 years with the exception of pT1-2 prostatic cancer Gleason score < 6, superficial bladder cancer, non-melanomatous skin cancer or carcinoma in situ of the cervix<br>11. Previous allogeneic tissue/solid organ transplant<br>12. Active infection requiring therapy<br>13. Surgery- or chemotherapy-related toxicity (non-hematological) not resolved to Grade 1 with the exception of alopecia, fatigue, neuropathy, and lack of appetite /nausea<br>14. Pregnant or breastfeeding, or expecting to conceive or father children within the projected duration of the trial, starting with the screening visit through 120 days after the last infusion of study treatment<br>15. Participant will not be eligible if the participant is or has an immediate family member (e.g., spouse, parent/legal guardian, sibling or child) who is investigational site or Sponsor staff directly involved with this trial, unless prospective site Review Board approval is given allowing exception to this criterion for a specific participant
TD-FOREKNOW 2023	1. Age: 18 years to 70 years (Adult, Older Adult)<br>2. ECOG physical score status of 0 or 1 points<br>3. Expected survival time ≥ 12 weeks<br>4. Pathological diagnosis with StageIIIA-IIIB NSCLC	1. Patients with brain metastasis<br>2. Patients with autoimmune disease, or a history of autoimmune disease including but not limited to the following: autoimmune hepatitis, interstitial pneumonia,

	5. According to the eighth edition of the AJCC/UICC TNM staging system, patients was pathological diagnosed with Stage III-N2 clinically resectable NSCLC.<br>6. Patients with at least one evaluable or measurable lesion as per RECIST version 1.1 （CT scan length and diameter of tumor lesion≥10mm，CT scan of lymph node lesion was short diameter≥15mm）<br>7. Patients were newly diagnosed with non-small cell lung cancer, without radiotherapy, chemotherapy, surgery, or molecule-targeted treatment.<br>8. Patients must have enough cardiopulmonary function for the expected pulmonary resections for lung cancer.<br>9. The main organ function meets the following criteria: 1) blood routine: a. ANC ≥ 1.5×109/L; b. PLT ≥ 100×109/L; c. HB ≥ 90 g/L; 2) Blood biochemistry:TBIL ≤ 1.5×ULN;ALT、AST≤ 2.5×ULN;sCr≤1.5×ULN; 3) Blood coagulation: INR≤1.5×ULN and APTT≤1.5×ULN， endogenous creatinine clearance rate≥50ml/min (Cockcroft–Gault formula)<br>10. Pregnancy test (serum or urine) has to be performed for woman of childbearing age within 7 days before enrollment and the test result must be negative. They shall take appropriate methods for contraception during the study until 3 months after the last administration of study drug. For men, (previous surgical sterilization accepted), they shall agree to take appropriate methods of contraception during the study until 3 months after the last administration of study drug;<br>11. Patient has to voluntarily join the study and sign the informed consent form for the study.	uveitis, enteritis, hepatitis, pituitary inflammation, vasculitis, myocarditis, nephritis, hyperthyroidism, hypothyroidism, which can be included after hormone replacement therapy; subjects with childhood asthma having been completely alleviated and without any intervention or vitiligo in adulthood can be included.<br>3. Subjects who need medical intervention with bronchodilators cannot be included.<br>4. Subjects with congenital or acquired immunodeficiency such as HIV infection, active hepatitis B (HBV DNA ≥ 2000 IU/mL), hepatitis C (hepatitis C antibody is positive)<br>5. Subjects with a condition requiring other immunosuppressive medications before 14 days of study drug administration firstly, not including inhaled corticosteroids or physiological doses of systemic treatment with either corticosteroids (> 10 mg daily prednisone equivalents).<br>6. Has received a live vaccine within 4 weeks of planned start of study therapy.<br>7. Other malignancies have been diagnosed within 3 years prior to the first use of the study drug; patients with current or a history of pulmonary fibrosis, interstitial pneumonia, pneumoconiosis, radiologic pneumonia, drug-induced pneumonia, and severe impairment of lung function.<br>8. Patients with hypertension that is difficult to control (systolic blood pressure ≥140 mmHg and diastolic blood pressure ≥90 mmHg)<br>9. Patients with myocardial ischemia and myocardial infarction above class II (including QT interval prolongation, for man ≥ 450 ms, for woman ≥ 470 ms)

		10. Severe infection within 4 weeks before the first administration (such as intravenous drip of antibiotics, antifungal drugs, or antiviral drugs), or fever of unknown origin (> 38.5 °C) within 4 weeks before the first administration.<br>11. Allogeneic organ transplantation (except corneal transplantation) or allogeneic hematopoietic stem cell transplantation.<br>12. Pregnant or nursing women; patients with a history of hypersensitivity to any of the study drugs, similar drugs, or excipients.<br>13. Participated in other clinical trials within 4 weeks; patients with a history of drug or alcohol abuse.<br>14. The investigator believes that there are any conditions that may damage the subject or result in the subject being unable to meet or perform the research request.

Table S2 Search strategies.

Database	Search Strategy
**PubMed**	("Immune Checkpoint Inhibitors" OR "Immune Checkpoint Inhibitor" OR "Checkpoint Inhibitors" OR "Checkpoint Inhibitor" OR "Immune Checkpoint Blockers" OR "Checkpoint Blockers" OR "Immune Checkpoint Blockade" OR "Checkpoint Blockade" OR "Immune Checkpoint Inhibition" OR "Checkpoint Inhibition" OR "PD-L1 Inhibitors" OR "PD L1 Inhibitors" OR "PD-L1 Inhibitor" OR "PD L1 Inhibitor" OR "Programmed Death-Ligand 1 Inhibitors" OR "Programmed Death Ligand 1 Inhibitors" OR "PD-1/PD-L1 Blockade" OR "Blockade PD-1/PD-L1" OR "PD 1 PD L1 Blockade" OR "PD-1 Inhibitors" OR "PD 1 Inhibitors" OR "PD-1 Inhibitor" OR "PD-1 Inhibitor" OR "PD 1 Inhibitor" OR "Programmed Cell Death Protein 1 Inhibitor" OR "Programmed Cell Death Protein 1 Inhibitors" OR nivolumab OR Pembrolizumab OR Dostarlimab OR Durvalumab OR atezolizumab OR avelumab) AND ("Carcinoma, Non Small Cell Lung" OR "Carcinomas, Non-Small-Cell Lung" OR "Lung Carcinoma, Non-Small-Cell" OR "Lung Carcinomas, Non-Small-Cell" OR "Non-Small-Cell Lung Carcinomas" OR "Non-Small-Cell Lung Carcinoma" OR "Non Small Cell Lung Carcinoma" OR "Carcinoma, Non-Small Cell Lung" OR "Non-Small Cell Lung Carcinoma" OR "Non-Small Cell Lung Cancer" OR "Non-Small-Cell Lung Cancer" OR NSCLC OR "Nonsmall Cell Lung Cancer") AND (RCT OR random OR randomly OR randomized OR randomised OR randomization OR "Randomized Controlled Trial" OR "Randomized Clinical Trial")
**Embase**	(TITLE-ABS-KEY("Immune Checkpoint Inhibitors") OR TITLE-ABS-KEY("Immune Checkpoint Inhibitor") OR TITLE-ABS-KEY("Checkpoint Inhibitors") OR TITLE-ABS-KEY("Checkpoint Inhibitor") OR TITLE-ABS-KEY("Immune Checkpoint Blockers") OR TITLE-ABS-KEY("Checkpoint Blockers") OR TITLE-ABS-KEY("Immune Checkpoint Blockade") OR TITLE-ABS-KEY("Checkpoint Blockade") OR TITLE-ABS-KEY("Immune Checkpoint Inhibition") OR TITLE-ABS-KEY("Checkpoint Inhibition") OR TITLE-ABS-KEY("PD-L1 Inhibitors") OR TITLE-ABS-KEY("PD L1 Inhibitors") OR TITLE-ABS-KEY("PD-L1 Inhibitor") OR TITLE-ABS-KEY("PD L1 Inhibitor") OR TITLE-ABS-KEY("Programmed Death-Ligand 1 Inhibitors") OR TITLE-ABS-KEY("Programmed Death Ligand 1 Inhibitors") OR TITLE-ABS-KEY("PD-1/PD-L1 Blockade") OR TITLE-ABS-KEY("Blockade PD-1/PD-L1") OR TITLE-ABS-KEY("PD 1 PD L1 Blockade") OR TITLE-ABS-KEY("PD-1 Inhibitors") OR TITLE-ABS-KEY("PD 1 Inhibitors") OR TITLE-ABS-KEY("PD-1 Inhibitor") OR TITLE-ABS-KEY("PD-1 Inhibitor") OR TITLE-ABS-KEY("PD 1 Inhibitor") OR TITLE-ABS-KEY("Programmed Cell Death Protein 1 Inhibitor") OR TITLE-ABS-KEY("Programmed Cell Death Protein 1 Inhibitors") OR TITLE-ABS-KEY(nivolumab) OR TITLE-ABS-KEY(Pembrolizumab) OR TITLE-ABS-KEY(Dostarlimab) OR TITLE-ABS-KEY(Durvalumab) OR TITLE-ABS-KEY(Atezolizumab) OR TITLE-ABS-KEY(Avelumab)) AND (TITLE-ABS-KEY("Carcinoma, Non Small Cell Lung") OR TITLE-ABS-KEY("Carcinomas, Non-Small-Cell Lung") OR TITLE-ABS-KEY("Lung Carcinoma, Non-Small-Cell") OR TITLE-ABS-KEY("Lung Carcinomas, Non-Small-Cell") OR TITLE-ABS-KEY("Non-Small-Cell Lung Carcinomas") OR TITLE-ABS-KEY("Non-Small-Cell Lung Carcinoma") OR TITLE-ABS-KEY("Non Small Cell Lung Carcinoma") OR TITLE-ABS-KEY("Carcinoma, Non-Small Cell Lung") OR TITLE-ABS-KEY("Non-Small Cell Lung Carcinoma") OR TITLE-ABS-KEY("Non-Small Cell Lung Cancer") OR TITLE-ABS-KEY("Non-Small-Cell Lung Cancer") OR TITLE-ABS-KEY(NSCLC) OR TITLE-ABS-KEY("Nonsmall Cell Lung Cancer")) AND (TITLE-ABS-KEY(RCT) OR TITLE-ABS-KEY(random) OR TITLE-ABS-KEY(randomly) OR TITLE-ABS-KEY(randomized) OR TITLE-ABS-KEY(randomised)

	OR TITLE-ABS-KEY(randomization) OR TITLE-ABS-KEY("Randomized Controlled Trial") OR TITLE-ABS-KEY("Randomized Clinical Trial"))
**The Cochrane Library**	(("Immune Checkpoint Inhibitors"):ti,ab,kw OR ("Immune Checkpoint Inhibitor"):ti,ab,kw OR ("Checkpoint Inhibitors"):ti,ab,kw OR ("Checkpoint Inhibitor"):ti,ab,kw OR ("Immune Checkpoint Blockers"):ti,ab,kw OR ("Checkpoint Blockers"):ti,ab,kw OR ("Immune Checkpoint Blockade"):ti,ab,kw OR ("Checkpoint Blockade"):ti,ab,kw OR ("Immune Checkpoint Inhibition"):ti,ab,kw OR ("Checkpoint Inhibition"):ti,ab,kw OR ("PD-L1 Inhibitors"):ti,ab,kw OR ("PD L1 Inhibitors"):ti,ab,kw OR ("PD-L1 Inhibitor"):ti,ab,kw OR ("PD L1 Inhibitor"):ti,ab,kw OR ("Programmed Death-Ligand 1 Inhibitors"):ti,ab,kw OR ("Programmed Death Ligand 1 Inhibitors"):ti,ab,kw OR ("PD-1/PD-L1 Blockade"):ti,ab,kw OR ("Blockade PD-1/PD-L1"):ti,ab,kw OR ("PD 1 PD L1 Blockade"):ti,ab,kw OR ("PD-1 Inhibitors"):ti,ab,kw OR ("PD 1 Inhibitors"):ti,ab,kw OR ("PD-1 Inhibitor"):ti,ab,kw OR ("PD-1 Inhibitor"):ti,ab,kw OR ("PD 1 Inhibitor"):ti,ab,kw OR ("Programmed Cell Death Protein 1 Inhibitor"):ti,ab,kw OR ("Programmed Cell Death Protein 1 Inhibitors"):ti,ab,kw OR (Nivolumab):ti,ab,kw OR (Pembrolizumab):ti,ab,kw OR (Dostarlimab):ti,ab,kw OR (Durvalumab):ti,ab,kw OR (Atezolizumab):ti,ab,kw OR (Avelumab):ti,ab,kw) AND (("Carcinoma, Non Small Cell Lung"):ti,ab,kw OR ("Carcinomas, Non-Small-Cell Lung"):ti,ab,kw OR ("Lung Carcinoma, Non-Small-Cell"):ti,ab,kw OR ("Lung Carcinomas, Non-Small-Cell"):ti,ab,kw OR ("Non-Small-Cell Lung Carcinomas"):ti,ab,kw OR ("Non-Small-Cell Lung Carcinoma"):ti,ab,kw OR ("Non Small Cell Lung Carcinoma"):ti,ab,kw OR ("Carcinoma, Non-Small Cell Lung"):ti,ab,kw OR ("Non-Small Cell Lung Carcinoma"):ti,ab,kw OR ("Non-Small Cell Lung Cancer"):ti,ab,kw OR ("Non-Small-Cell Lung Cancer"):ti,ab,kw OR (NSCLC):ti,ab,kw OR ("Nonsmall Cell Lung Cancer"):ti,ab,kw) AND ((RCT):ti,ab,kw OR (random):ti,ab,kw OR (randomly):ti,ab,kw OR (randomized):ti,ab,kw OR (randomised):ti,ab,kw OR (randomization):ti,ab,kw OR ("Randomized Controlled Trial"):ti,ab,kw OR ("Randomized Clinical Trial"):ti,ab,kw)

Table S3 Additional baseline characteristics of included studies.

Study	ECOG performace status score, no. (%)		Smoking status, no. (%)		Race or ethic group, no. (%)		Disease stage, no. (%)		EGFR mutation status, no. (%)		ALK translocation, no .(%)	
	IG	CG	IG	CG	IG	CG	IG	CG	IG	CG	IG	CG
PEARLS/KEYNOTE-091 2022	0: 380 (64)<br>1: 210 (36)	0: 343 (58)<br>1: 244 (42)	75 (13) Current, 428 (73) Former, 87 (15) Never	90 (15) Current, 431 (73) Former, 66 (11) Never	1 (<1) American Indian or Alaskan Native, 107 (18) Asian, 4 (1) Multiple, 6 (1) Other, 450 (76) White, 22 (4) Missing	107 (18) Asian, 3 (1) Black or African American, 1 (<1) Multiple, 2 (<1) Other, 455 (78) White, 19 (3) Missing	IB: 84 (14)<br>II: 329 (56)<br>IIIA: 177 (30)	IB: 84 (14)<br>II: 329 (56)<br>IIIA: 177 (30)<br>IV: 2 (<1%)*	39 (7) Yes, 218 (37) No, 333 (56) Unknown	34 (6) Yes, 216 (37) No, 337 (57) Unknown	7 (1) Yes, 226 (38) No, 357 (61) Unknown	7 (1) Yes, 190 (32) No, 390 (66) Unknown
CheckMate 816 2022	0: 124 (69.3)<br>1: 55 (30.7)	0: 117 (65.4)<br>1: 62 (34.6)	19 (10.6) Never smoked, 160 (89.4) Current or former smoker	20 (11.2) Never smoked, 158 (88.3) Current or former smoker	NA	NA	IB or II: 65 (36.3)<br>IIIA: 113 (63.1)	IB or II: 62 (34.6)<br>IIIA: 115 (64.2)	NA	NA	NA	NA
NADIM II 2023	0: 31 (54)<br>1: 26 (46)	0: 16 (55)<br>1: 13 (45)	30 (53) Current, 22 (39) Former, 5 (9) Never	21 (72) Current, 8 (28) Former	NA	NA	T1N2M0: 12 (21)<br>T2N2M0: 16 (28)<br>T3N1M0: 2 (4)<br>T3N2M0: 13 (23)<br>T4N0M0: 6 (11)<br>T4N1M0: 8 (14)	T1N2M0: 4 (14)<br>T2N2M0: 7 (24)<br>T3N1M0: 1 (3)<br>T3N2M0: 5 (17)<br>T4N0M0: 9 (31)<br>T4N1M0: 3 (10)	NA	NA	NA	NA
KEYNOTE-671 2023	0: 253 (63.7)<br>1: 144 (36.3)	0: 246 (61.5)<br>1: 154 (38.5)	96 (24.2) Current, 247 (62.2) Former, 54 (13.6) Never	103 (25.8) Current, 250 (62.5) Former, 47 (11.8) Never	1 (0.3) American Indian or Alaska Native, 124 (31.2) Asian, 6 (1.5) Black, 3 (0.8) Multiple, 250 (63.0) White, 13 (3.3) Missing	125 (31.2) Asian, 10 (2.5) Black, 10 (2.5) Multiple, 239 (59.8) White, 16 (4.0) Missing	II: 118 (29.7)<br>IIIA: 217 (54.7)<br>IIIB: 62 (15.6)	II: 121 (30.2)<br>IIIA: 225 (56.2)<br>IIIB: 54 (13.5)	14 (3.5) Yes, 111 (28.0) No, 272 (68.5) Unknown	19 (4.8) Yes, 127 (31.8) No, 254 (63.5) Unknown	12 (3.0) Yes, 104 (26.2) No, 281 (70.8) Unknown	9 (2.2) Yes, 133 (33.2) No, 258 (64.5) Unknown

IMpower010 2021	0: 273 (54) 1: 232 (46) 2: 232 (46)	0: 283 (57) 1: 214 (43) 2: 1 (<1)	76 (15) Current, 317 (63) Previous, 114 (23) Never	86 (17%) Current, 304 (61%) Previous, 108 (22%) Never	362 (71) White; 130 (26) Asian; 5 (1) Black or African American; 1 (<1) Native Hawaiian or other Pacific Islander; 9 (2) Unknown	376 (76) White; 112 (23) Asian; 1 (<1) Black or African American; 1 (<1) Native Hawaiian or other Pacific Islander; 1 (<1)Multiple; 7 (1) Unknown	IB:65 (13) IIA: 147 (29) IIB: 90 (18) IIIA: 205 (40)	IB: 58 (12) IIA: 148 (30) IIB: 84 (17) IIIA: 208 (42)	53 (10) Yes, 261 (52) No, 193 (38) Unknown	64 (13) Yes, 266 (53) No, 168 (34) Unknown	15 (3) Yes, 280 (55) No, 212 (42) Unknown	18 (4%) Yes, 294 (59%) No, 186 (37%) Unknown
NEOTORCH 2023	NA	NA	NA	NA	NA	NA	NA	NA	202 (100) No	202 (100) No	202 (100) No	202 (100) No
TD-FOREKNOW 2023	0: 41 (95.3) 1: 2 (4.7)	0: 43 (95.6) 1: 2 (4.4)	12 (27.9) Nonsmoker, 31 (72.1) Former or current smoker	8 (17.8) Nonsmoker, 37 (82.2) Former or current smoker	NA	NA	IIIA: 30 (69.8) IIIB: 13 (30.2)	IIIA: 36 (80.0) IIIB: 9 (20.0)	NA	NA	NA	NA

*Metastatic disease was discovered soon after randomization upon review of additional protocol-required screening CT images in one participant and of a non-protocol-required lumbar MRI assessment in the second participant; because of ineligibility, both participants were discontinued from active study treatment before the first administration but were included in the intention-to-treat population. ALK, anaplastic lymphoma kinase; CG, control group; ECOG, Eastern Cooperative Oncology Group; EGFR, epidermal growth factor receptor; IG, intervention group; NA, not available.

Table S4 Treatment regimens from the randomized controlled trials included in this systematic review and meta-analysis.

Study	Treatment regimes
CheckMate 816 2022	Before undergoing surgery, patients were randomized to receive nivolumab (360 mg) plus platinum-doublet chemotherapy or platinum-doublet chemotherapy alone, every 3 weeks for three cycles. Surgery was planned to be performed within 6 weeks after the end of NAT.
IMpower010 2021	Patients received AT in up to four 21-day cycles with cisplatin 75 mg/m² intravenously on day 1 of each cycle plus vinorelbine 30 mg/m² intravenously on days 1 and 8; docetaxel 75 mg/m² intravenously on day 1; gemcitabine 1250 mg/m² intravenously on days 1 and 8; or pemetrexed 500 mg/m² intravenously on day 1 for patients with non-squamous NSCLC. Additionally, patients received atezolizumab intravenously (1200 mg) on day 1 of each 21-day cycle for up to 16 cycles or best supportive care (observation and regular scans).
KEYNOTE-671 2023	Participants received NAT with four cycles of pembrolizumab (200mg) or placebo intravenously once every 3 weeks added to neoadjuvant chemotherapy with cisplatin and gemcitabine for squamous histologic features or cisplatin and pemetrexed for non-squamous histologic features. Then surgery should be performed within 20 weeks after the first dose of pembrolizumab. Adjuvant therapy was performed with pembrolizumab (200 mg) or placebo intravenously once every 3 weeks for up to 13 cycles.
NADIM II 2023	Patients received nivolumab (360 mg), paclitaxel (200 mg per square meter of body-surface area) plus carboplatin (area under the concentration–time curve, 5 mg per milliliter per minute), or paclitaxel plus carboplatin at the same doses as NAT every 21 days. Surgery should be performed within 3 to 4 weeks after day 21 of the third cycle of NAT. Intervention group patients with R0 resections (no residual tumor according to the International Association for the Study of Lung Cancer R0 criteria) received AT with nivolumab (480 mg) once every 4 weeks for 6 months.
NEOTORCH 2023	Participants received 3 cycles of NAT with toripalimab (240 mg) intravenously plus platinum-based doublet drug chemotherapy or placebo plus platinum-based doublet drug chemotherapy before undergoing radical surgery within 4-6 weeks after the third cycle. Thirty days after surgery, patients received one cycle of AT with platinum-based doublet drug chemotherapy with or without toripalimab (240 mg) intravenously. Patients underwent consolidation treatment with toripalimab (240 mg) intravenously or placebo, each cycle every 3 weeks for a total of 13 cycles. The AT was scheduled according to the presence of adjuvant radiotherapy.
PEARLS/ KEYNOTE-091 2022	After surgical resection, patients received pembrolizumab (200 mg) or saline placebo administered intravenously once every 3 weeks until completion of 18 administrations or other reasons. Adjuvant chemotherapy was performed when recommended per guidelines.
TD-FOREKNOW 2023	Camrelizumab (200 mg) was administered intravenously on 1 day of each 3-week cycle for 3 cycles before surgical resection. Additionally, NAT was administered with nab-paclitaxel (130 mg/m² intravenously on days 1 and 8) and platinum (cisplatin, 75 mg/m²; carboplatin, area under the curve, 5; or nedaplatin, 100 mg/m² intravenously on day 1) every 3 weeks for 3 cycles. Surgery was planned 4 to 6 weeks after the completion of neoadjuvant treatment.

AT, adjuvant therapy; NAT, neoadjuvant therapy; NSCLC, non-small cell lung cancer.

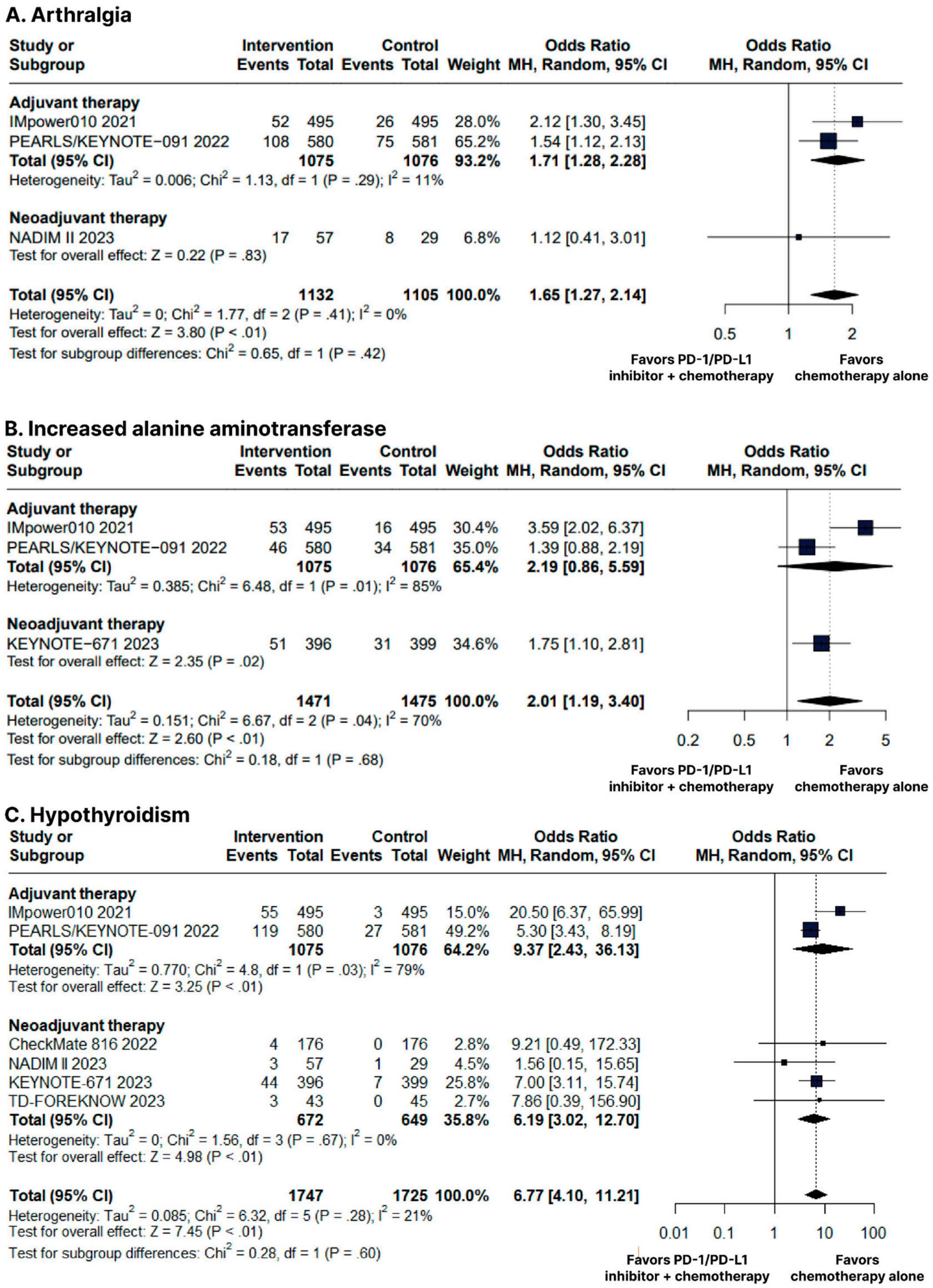

Supplementary Figure S1 Any grade of adverse events. **A** Arthralgia. **B** Increased alanine aminotransferase. **C** Hypothyroidism. Comparison between programmed cell death protein 1 (PD-1)/programmed death-ligand 1 (PD-L1) inhibitors plus chemotherapy and chemotherapy alone in

patients with resectable stage non-small cell lung cancer. CI, confidence interval; MH, Mantel–Haenszel.

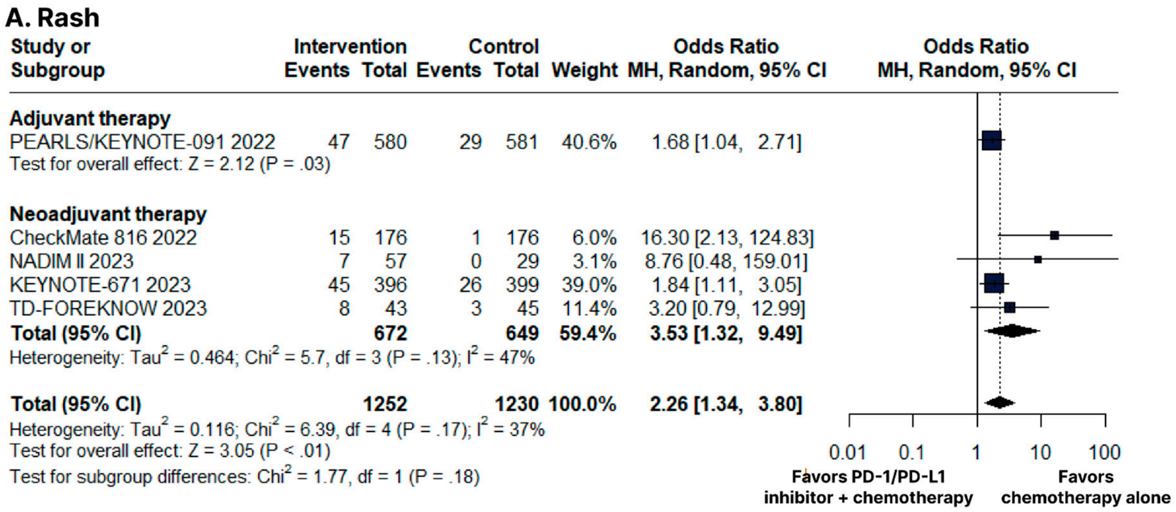

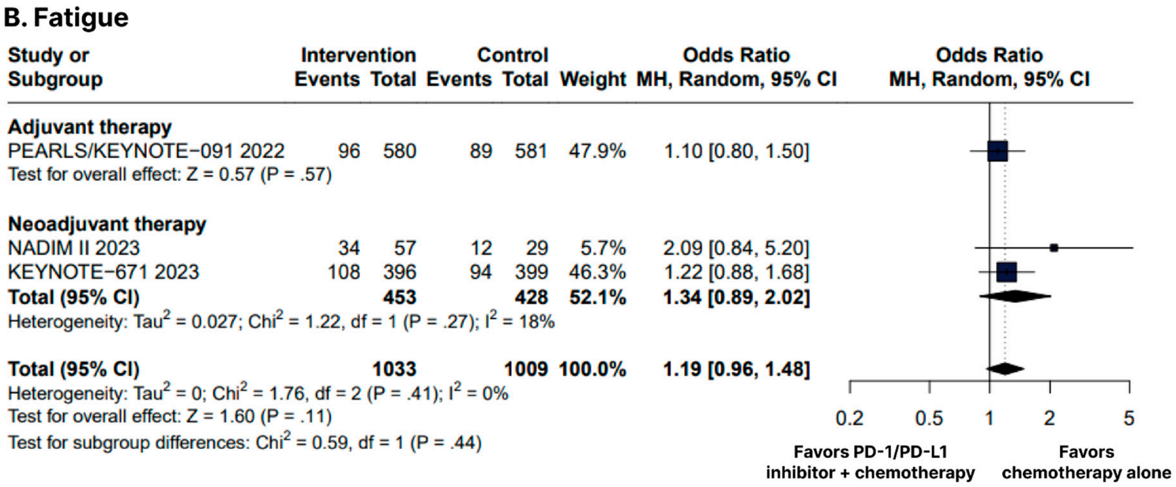

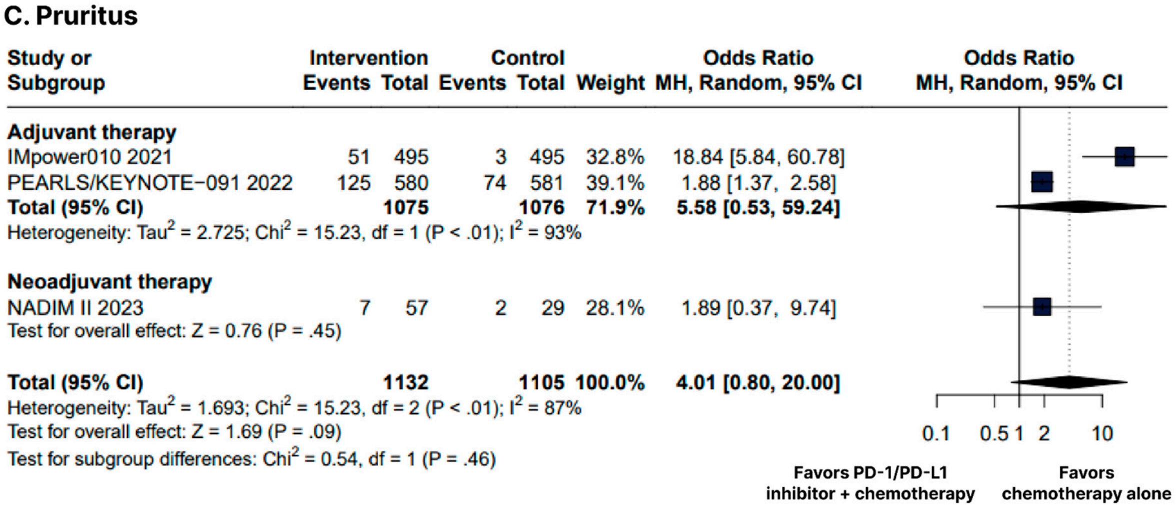

Supplementary Figure S2 Any grade of adverse events. **A** Rash. **B** Fatigue. **C** Pruritus. Comparison between programmed cell death protein 1 (PD-1)/programmed death-ligand 1 (PD-L1) inhibitors plus chemotherapy and chemotherapy alone in patients with resectable stage non-small cell lung cancer. CI, confidence interval; MH, Mantel–Haenszel.

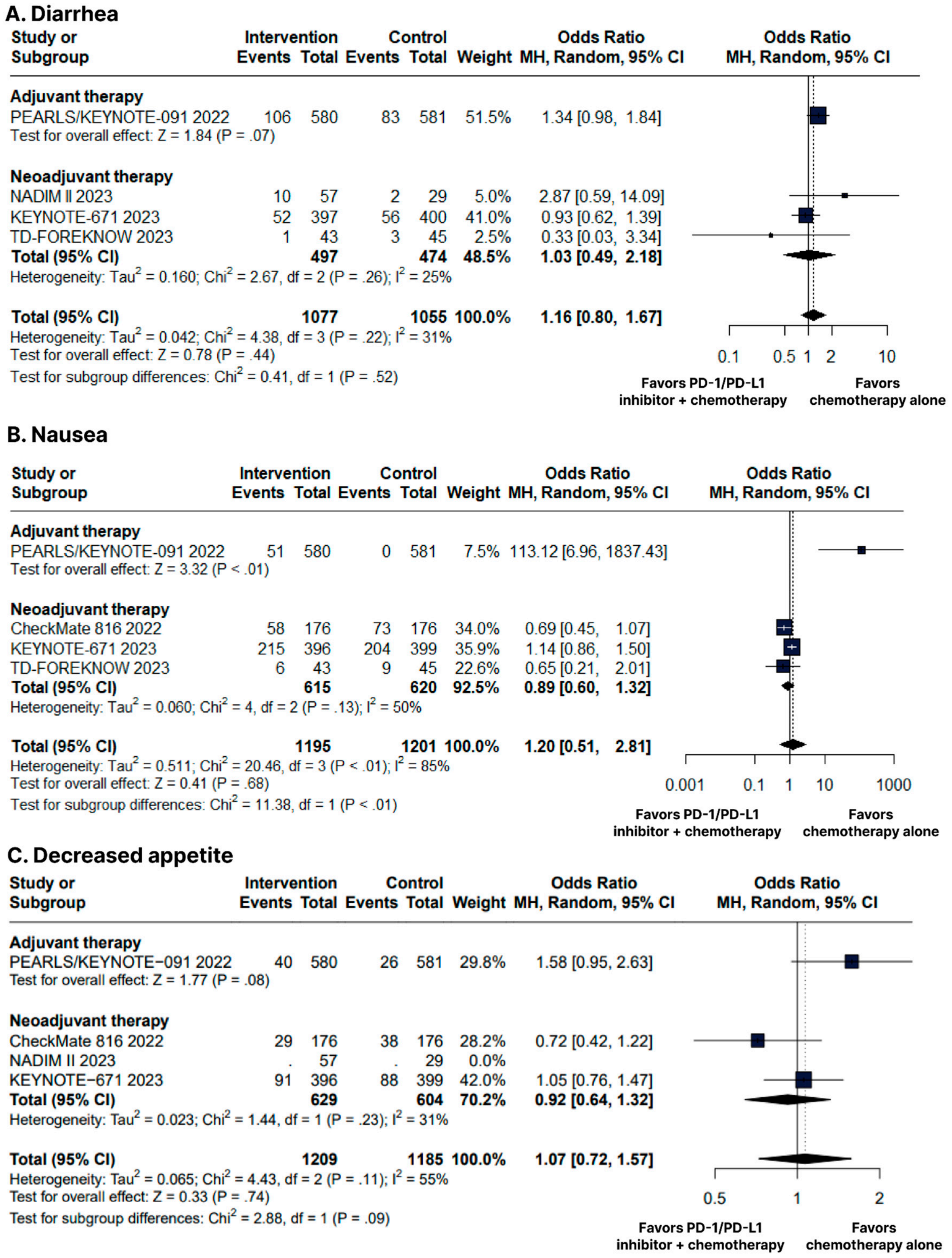

Supplementary Figure S3 Any grade of adverse events. **A** Diarrhea. **B** Nausea. **C** Decreased appetite. Comparison between programmed cell death protein 1 (PD-1)/programmed death-ligand 1 (PD-L1) inhibitors plus chemotherapy and chemotherapy alone in patients with resectable stage non-small cell lung cancer. CI, confidence interval; MH, Mantel–Haenszel.

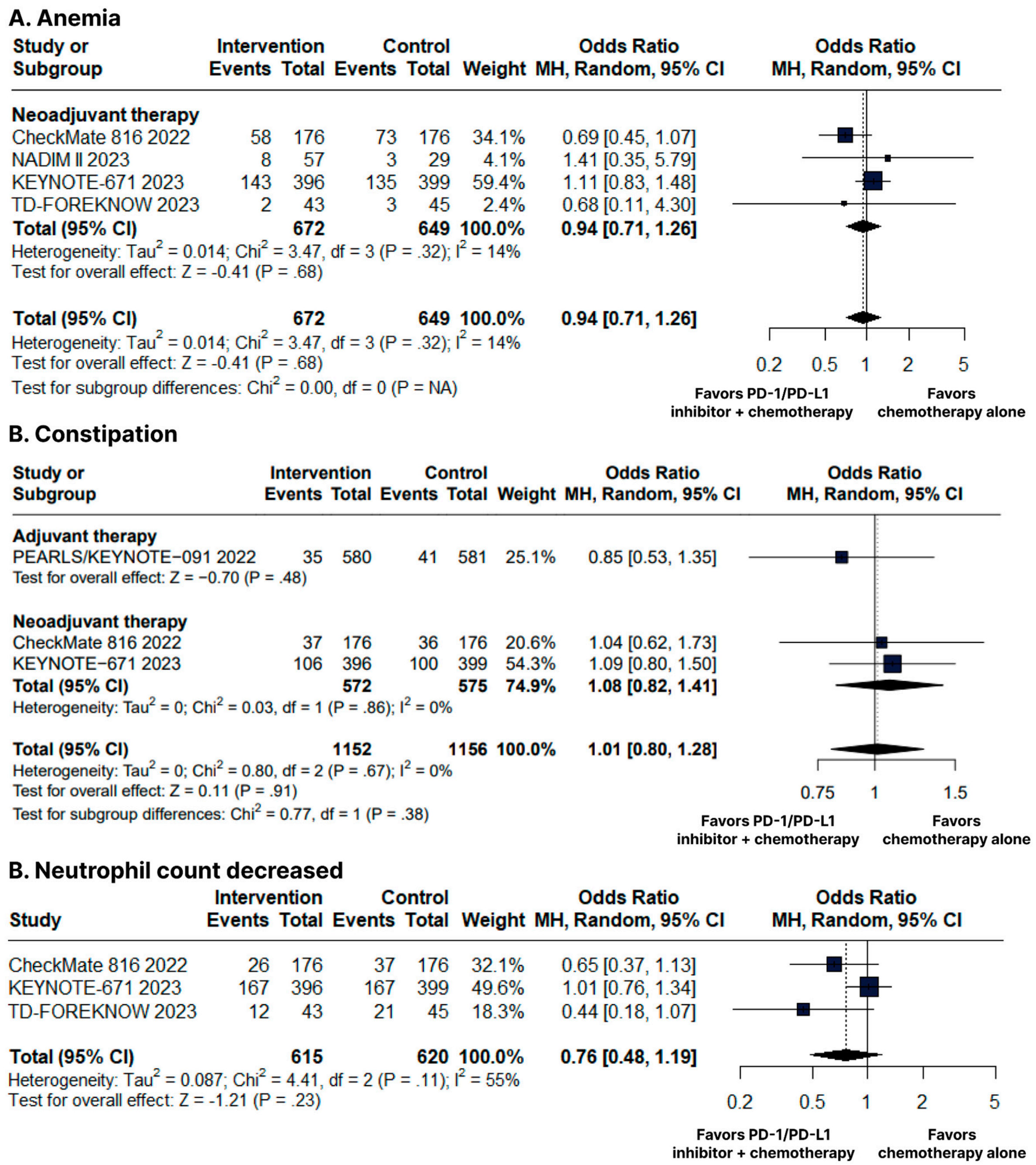

Supplementary Figure S4 Any grade of adverse events. **A** Anemia. **B** Constipation. **C** Neutrophil count decreased. Comparison between programmed cell death protein 1 (PD-1)/programmed death-ligand 1 (PD-L1) inhibitors plus chemotherapy and chemotherapy alone in patients with resectable stage non-small cell lung cancer. CI, confidence interval; MH, Mantel–Haenszel.

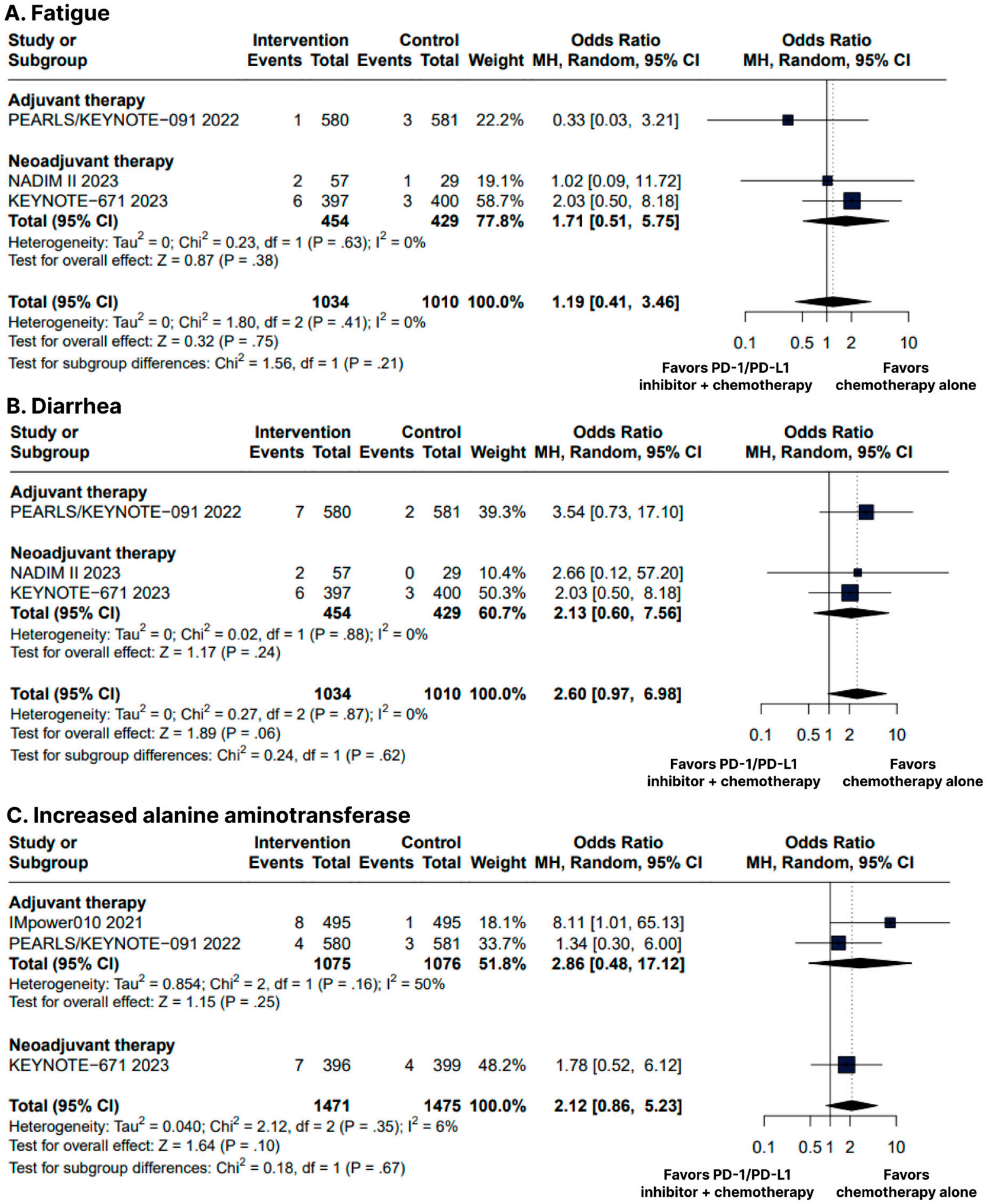

Supplementary Figure S5 Grade ≥3 adverse events. **A** Fatigue. **B** Diarrhea. **C** Increased alanine aminotransferase. Comparison between programmed cell death protein 1 (PD-1)/programmed death-ligand 1 (PD-L1) inhibitors plus chemotherapy and chemotherapy alone in patients with resectable stage non-small cell lung cancer. CI, confidence interval; MH, Mantel–Haenszel.

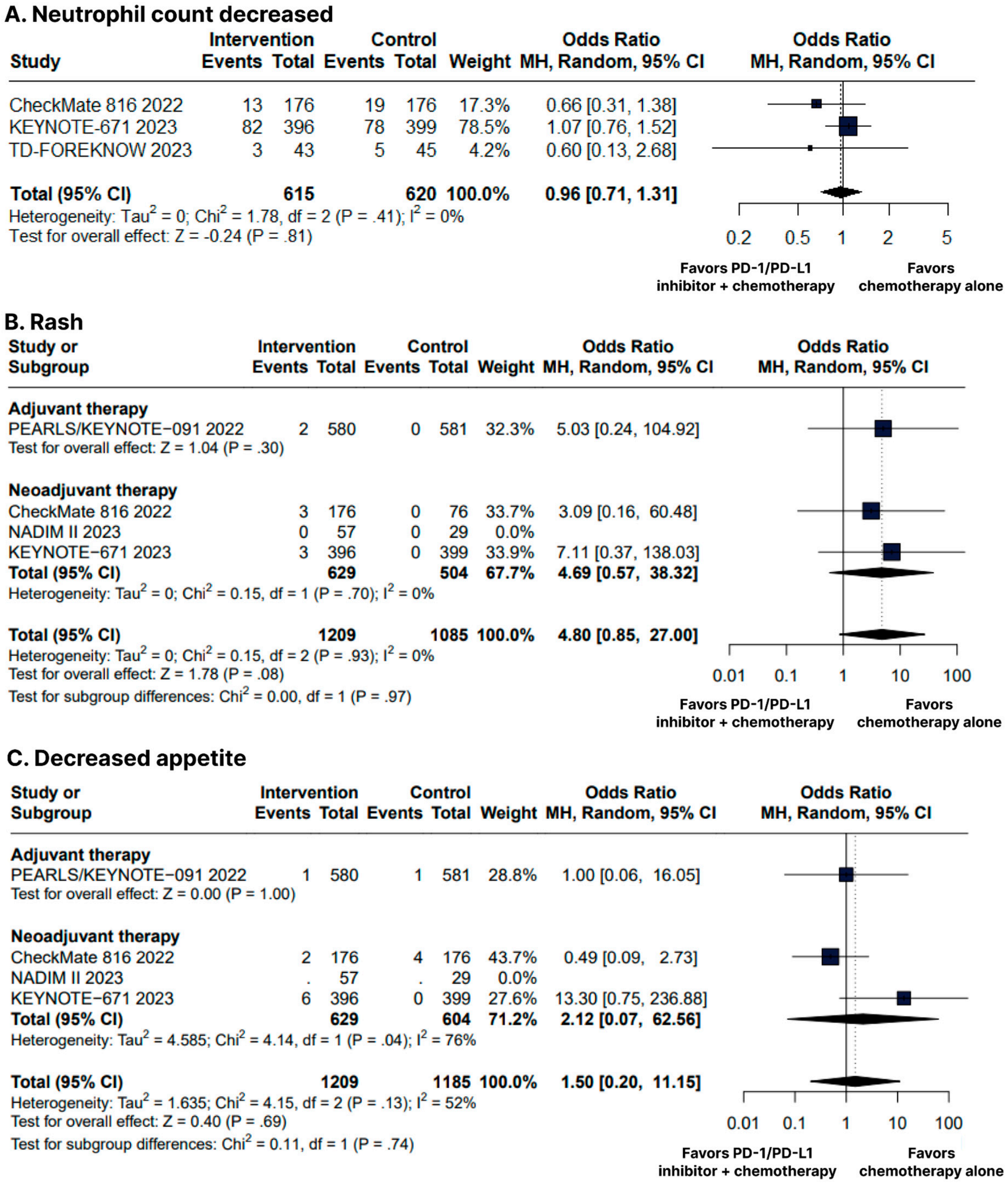

Supplementary Figure S6 Grade ≥3 adverse events. **A** Neutrophil count decreased. **B** Rash. **C** Decreased appetite. Comparison between programmed cell death protein 1 (PD-1)/programmed death-ligand 1 (PD-L1) inhibitors plus chemotherapy and chemotherapy alone in patients with resectable stage non-small cell lung cancer. CI, confidence interval; MH, Mantel–Haenszel.

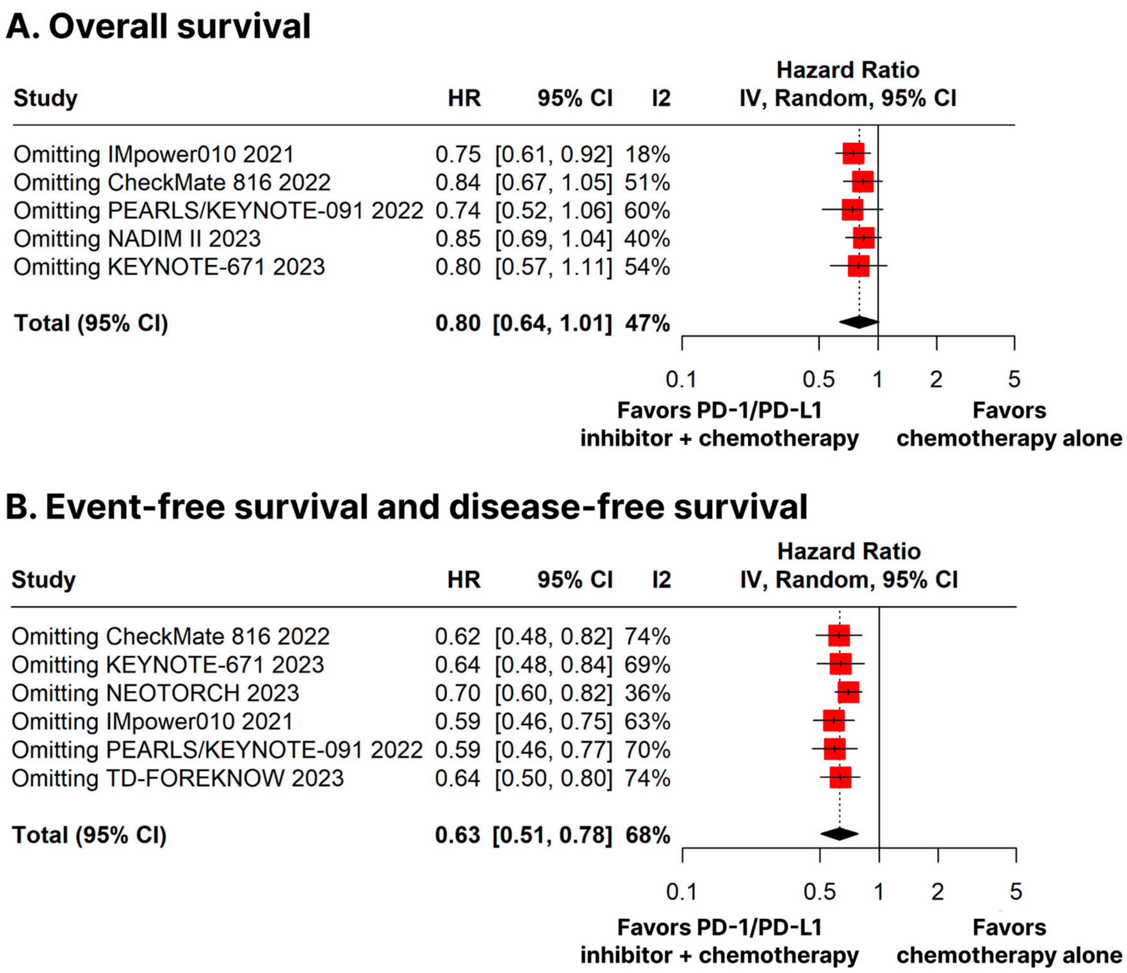

Supplementary Figure S7 Leave-one-out sensitivity analyses. **A** Overall survival. **B** Event-free survival and disease-free survival. CI, confidence interval; HR, hazard ratio; IV, inverse variance; PD-1, programmed cell death protein 1; PD-L1, programmed death-ligand 1.
